# Patient preferences for ambulatory blood pressure monitoring devices: Wrist-type or arm-type?

**DOI:** 10.1371/journal.pone.0255871

**Published:** 2021-08-09

**Authors:** Wei-wei Zeng, Sze Wa Chan, Brian Tomlinson

**Affiliations:** 1 Shenzhen Baoan Women’s and Children’s Hospital, Jinan University, Shenzhen, Guangdong Province, China; 2 School of Health Sciences, Caritas Institute of Higher Education, Tseung Kwan O, Hong Kong; 3 Faculty of Medicine, Macau University of Science and Technology, Taipa, Macau, China; 4 Department of Medicine & Therapeutics, The Chinese University of Hong Kong, Prince of Wales Hospital, Shatin, Hong Kong; International University of Health and Welfare, School of Medicine, JAPAN

## Abstract

**Background:**

Ambulatory blood pressure monitoring (ABPM) is important in evaluating average 24-hour blood pressure (BP) levels, circadian rhythm, sleeping BP and BP variability but many patients are reluctant to use standard ABPM devices.

**Methods:**

We compared two validated ABPM devices, the BPro tonometric wrist monitor and the A&D TM-2430 oscillometric upper arm monitor, for agreement of recordings and acceptability in 37 hypertensive patients (aged 55±9 years).

**Results:**

Successful BP measurements were less frequent with the wrist-type than the arm-type device during the sleeping (66.3% vs. 92.9%, *P* <0.01) and awake periods (56.2% vs. 86.5%, *P* <0.01). Comparable paired readings showed no significant difference in systolic BP but diastolic BP (DBP) values were higher with the wrist compared to the arm monitor (24-hour 89±13 vs. 85±14 mmHg, *P* <0.01) with similar differences awake and sleeping. Bland-Altman analysis showed some large discrepancies between individual arm and wrist monitor measurements. More patients found the wrist monitor more comfortable to use than the arm monitor.

**Conclusions:**

Despite the difference in individual BP measurements and the systematic overestimation of DBP values with the BPro device, wrist monitors with good patient acceptability may be useful to facilitate ABPM in some patients to provide additional information about cardiovascular risk and response to antihypertensive therapies.

## Introduction

Ambulatory blood pressure monitoring (ABPM) is recommended in recent hypertension guidelines to confirm the diagnosis of hypertension, to identify white-coat and masked hypertension, and to monitor blood pressure (BP) control [1–3]. It also provides important information on the variability of BP during 24 hours [4], the morning surge and the pattern of nocturnal dipping which have been associated with target organ damage and cardiovascular disease (CVD) prognosis [5]. In recent years, measures of BP variability, particularly the visit-to-visit variability and to a lesser extent the variability on ABPM, have been found to be strong predictors of stroke and other vascular events independent of mean clinic or ABPM measurements [6].

Home BP monitoring (HBPM) using validated automatic devices is also recommended and has the particular advantage of improving adherence with treatment regimens [1, 2, 7]. Elevated sleep-time BP has been reported in some studies to be a better predictor of CVD risk than either the awake or 24-hour mean BP [8]. It is difficult to measure sleep-time BP with most HBPM devices so that intermittent ABPM may be useful in addition to HBPM and could help to guide bedtime hypertension chronotherapy [9].

The arm-cuff ABPM was recommended as a standard ABPM device and it has been used widely since the noninvasive ABPM was introduced [10]. However, many patients find it uncomfortable and it often disturbs sleep so patients may be reluctant to undergo repeated ABPM sessions. The wrist type, cuff-less ABPM has also been introduced and widely used. Currently, there are several devices available that have been validated and are recommended for patient use. A watch like wrist type ABPM, called BPro (HealthSTATS International, Singapore), was validated in 2008 [11], according to the European Society of Hypertension (ESH) protocol [12] and the Association for the Advancement of Medical Instrumentation (AAMI) standard [13]. This wrist monitor measures ambulatory 24 hour BP with an arterial tonometry sensor which is placed over the radial artery to capture the radial artery pulse wave form.

Some studies have compared this device with other conventional arm-cuff ABPM devices including the A&D TM-2430 in complicated hypertensive patients [14], the A&D TM 2431 in normotensive and prehypertensive volunteers [15], the A&D TM 2421 in patients with diabetes [16] and Spacelabs 90207 in hypertensive patients [17].

In the present study, we compared the BPro wrist type ABPM with the A&D TM 2430 upper arm ABPM (A&D Company Limited, Tokyo, Japan), which was validated according to the Association for the Advancement of Medical Instrumentation (AAMI) and British Hypertension Society protocols (grade A/A) and recommended for clinical use in 1998 [18]. We evaluated the agreement of recordings and acceptability for use between these two validated devices in patients with mild to moderate essential hypertension.

## Materials and methods

### Subjects

From January 2013 to June 2014, consecutive suitable Chinese patients with mild to moderate essential hypertension who attended the outpatient clinics in the Prince of Wales Hospital, Hong Kong were invited to enter the study. Mild to moderate essential hypertension was defined as sitting clinic systolic BP (SBP) from 140 to 169 mmHg or sitting clinic diastolic BP (DBP) from 90 to 109 mmHg (130–169 mmHg or 80–109 mmHg for patients with diabetes mellitus). According to the European Society of Hypertension (ESH) BP device validation protocol (12), ≥33 subjects were required to gain a statistical power of 90% (two-sided alpha = 0.05). A total of 37 hypertensive patients with mean (±SD) age 55±9 years (range 18–79 years) completed this comparison study and the statistical power was 99% with a two-sided alpha of 0.05. Informed consent was obtained from each patient before undertaking any study related activity. This study was a sub-study of the main study which was approved by the Joint Chinese University of Hong Kong-New Teritories East Cluster Clinical Research Ethics Committee with a reference number of CRE-2011.616-T.

### Blood pressure measurements

Before starting the wrist and arm ABPM recordings, the clinic seated BP was measured in both arms using an automatic arm monitor (GE DINAMAP Pro 100, UK) to ensure there was no difference in BP between arms. The average BP was calculated from three clinic seated BP measurements (GE DINAMAP Pro 100, UK) taken at 1 minute intervals. This average BP was entered into the wrist ABPM device before starting the 24-hour ABPM recordings. The tonometer in the wrist strap of the BPro was placed over the left radial artery. The arm monitor cuff was applied to the right upper arm.

The wrist monitor was set to measure BP every 15 minutes during the 24-hour period and the arm monitor was set to measure BP at 15 minute intervals during 07:00~22:00 and every 1 hour during 22:00~07:00. Subjects were requested to keep the arm still and parallel to the trunk when the arm-cuff was inflated and to keep the left wrist at heart level when the wrist-type measurements were being performed. The patients were encouraged to continue their usual daily activities but not to engage in vigorous physical exercise such as running, climbing, or playing sports. A daily activity record form was given to each patient. At the end of the 24-hour ABPM, each patient was asked to choose which device they would prefer if they were having ABPM in the future. ABPM recordings were excluded from the analyses if the percentage of valid readings was below 70% or if readings were lacking for an interval more than 2 hours or if there were less than 14 readings during the daytime period, or less than 7 readings during the nighttime period.

### Statistical analysis

ABPM profiles obtained by wrist and arm devices were edited for measurement errors and outliers according to conventional criteria; SBP readings >250 or <70 mmHg, DBP readings >150 or <40 mmHg, pulse pressure (PP; difference between SBP and DBP) values >150 or <20 mmHg, and readings with a change of ≥50 mmHg in DBP between consecutive readings were eliminated. The valid paired readings measured within ≤5 minutes of each other and having a difference in heart rate ≤5 beats/minute by the wrist and arm devices were compared. The 24-hour BP was calculated for each device as the average of all paired readings obtained during the 24-hour ABPM. Awake and asleep BP values were calculated as the average of all paired readings measured during the hours of daytime activity or nighttime sleep, respectively. The daytime activity and nighttime sleep period were based on the information obtained from the patient’s daily activity record form.

All statistical analyses were carried out using SPSS software, version 18.0 (SPSS Inc., Chicago IL, USA). The individual BP values obtained using the two monitors were evaluated as mean ± standard deviation (SD). The group values and proportions were compatible with a normal distribution. The group means were compared using paired Student’s t-tests. The group proportions were compared using the χx^2^-test. Bland–Altman plots were used for the comparison of the readings from the two monitors. The correlations between the values from the two monitors were evaluated by Pearson’s correlation coefficients and intra-class correlation (ICC) agreements.

## Results

Thirty-seven participants completed the study. The wrist monitor was preferred by 27 patients as the patients reported that it caused fewer disturbances than the arm monitor, especially during sleep, and overall it was more comfortable to use, while the other 10 patients preferred the arm monitor as they had discomfort in the wrist area when using the wrist monitor. Another reason for preferring the wrist monitor was that it is placed on the radial artery and can measure BP without removing clothing for a cuff application. As shown in Table 1, the mean age of the 37 patients was 55±9 years and the mean body mass index was 25.2±3.0 kg/m^2^. There were 14 male (38%) patients in this study. There were 14 patients taking amlodipine as antihypertensive treatment and the other patients were on no antihypertensive treatment. Eight patients were on anti-hyperglycemic treatment for type 2 diabetes mellitus.

**Table 1:** Demographic and clinic characteristics of study subjects.

Characteristics	Values
N	37
Age, years	55±9 (range 18–79)
Sex, male %	14 (38%)
Body mass index, kg/m^2^	25.2±3.0
Clinic seated SBP, mmHg	139±14
Clinic seated DBP, mmHg	85±11
Diabetes mellitus	8 (22%)
With amlodipine treatment	14 (38%)

Data were presented as mean ± SD or n (%).

DBP = diastolic blood pressure, SBP = systolic blood pressure.

The success rates of the BP measurements according to the wrist-type and the arm-type devices are shown in Table 2. The mean total number of successful readings for the 24-hour BP was greater for the arm-type than for the wrist-type device (57±13 vs. 51±18, *P* <0.01). There were a greater (*P* <0.01) number of successful readings for the sleeping period using the wrist-type device (21±8 readings) compared with the arm-type device (10.5±4.8 readings). The frequency of attempted readings was greater with the wrist-type than the arm-type device during 22:00~07:00 so the success rate was lower with the wrist-type device during the sleeping period (66.3% vs. 92.9%, *P* <0.01) and the awake period (56.2% vs. 86.5%, *P* <0.01).

**Table 2:** Numbers and percentages of successful blood pressure readings by the two monitors (n = 37).

	Arm-type			Wrist-type				
	Number of successful measurements	Number of measurements	Success rate (%)	Number of successful measurements	Number of measurements	Success rate (%)	*P1*-value	*P2*-value
24 hour	57±13	65±13	88.1	51±18	87±21	58.9	<0.01	<0.01
Awake	48±11	55±11	86.5	33±11	59±12	56.2	<0.01	<0.01
Sleeping	10.5±4.8	10.8±4.8	92.9	21±8	31±4	66.3	<0.01	<0.01

*P1*-value shows the difference of success rates between the two types of monitor.

*P2*-value shows the difference of number of successful measurements between the two types of monitor.

The average BP levels are shown in Table 3. From 912 readings (681 awake, 231 asleep readings) that could be compared between monitors, the average 24-hour DBP, awake DBP and sleeping DBP were significantly lower using the arm type device compared with the wrist type device (85±14 vs. 89±13 mmHg, *P* <0.01; 88±14 vs. 91±13 mmHg, *P* <0.01; 79±13 vs 83±12 mmHg, *P* <0.01). There was no significant difference in the SBP measurements between the two devices. Pearson’s correlations and intra-class correlations were significant for all comparisons. A regression analysis was performed to identify if any baseline factors such as age, sex, body weight, BMI, 24-hour SBP and DBP measured by the two devices were related to the discordance and it was found there were only the 24-hour SBP values measured by the two devices which were related to the differences in SBP between the two devices. None of the factors tested were significantly related to the differences in DBP between the two devices.

**Table 3:** Comparison of average BP levels provided by the two monitors (n = 37).

	Readings (n)	Arm-type	Wrist-type	Difference	*P1*-value	Pearson’s correlation coefficients	*P2*-value	ICC (95% CI)	*P3*-value
24 hour SBP, mmHg	912	142±16	141±17	0.7±19.8	0.24	0.41	<0.01	0.41 (0.36–0.47)	<0.01
24 hour DBP, mmHg	912	85±14	89±13	-3.9±13.5	<0.01	0.51	<0.01	0.51 (0.46–0.56)	<0.01
Awake SBP, mmHg	681	142±17	141±17	1.1±19.1	0.13	0.35	<0.01	0.35 (0.29–0.42)	<0.01
Awake DBP, mmHg	681	88±14	91±13	-3.7±13.9	<0.01	0.46	<0.01	0.46 (0.40–0.52)	<0.01
Sleeping SBP, mmHg	231	129±18	129±15	-0.3±19.4	0.80	0.33	<0.01	0.32 (0.20–0.43)	<0.01
Sleeping DBP, mmHg	231	79±13	83±12	-4.7±12.5	<0.01	0.51	<0.01	0.51 (0.41–0.60)	<0.01

DBP = diastolic blood pressure, ICC = intra-class correlation, SBP = systolic blood pressure.

*P1*-value shows the difference of BP values between the two types of monitor.

*P2*-value shows the difference from Pearson’s correlation coefficients.

*P3*-value shows the difference from ICC agreement with 0.5 as a reference.

### Bland-Altman analysis

**Fig 1:**
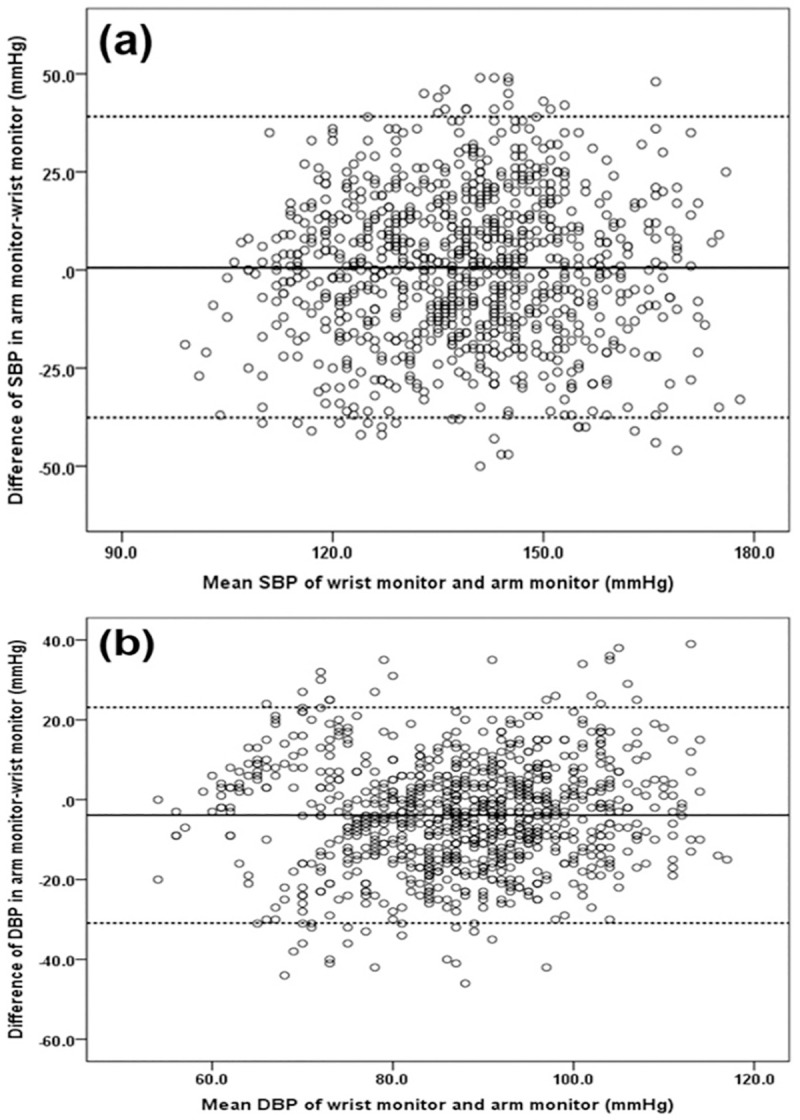
Bland–Altman plots presenting the mean and the difference in blood pressure measurements (mmHg) between the wrist and arm monitors over 24 hours for SBP (a) and DBP (b). The solid black horizontal line represents the mean difference and the dotted horizontal lines represent 2SD above and below the mean. There was a mean difference of +0.7 mmHg and a 2SD difference between the arm monitor and the wrist monitor of -37.7 to 39.1 mmHg for SBP and a mean difference of -3.9 mmHg and a 2SD difference of -30.9 to 23.1 mmHg for DBP. DBP = diastolic blood pressure, SBP = systolic blood pressure.

**Fig 2:**
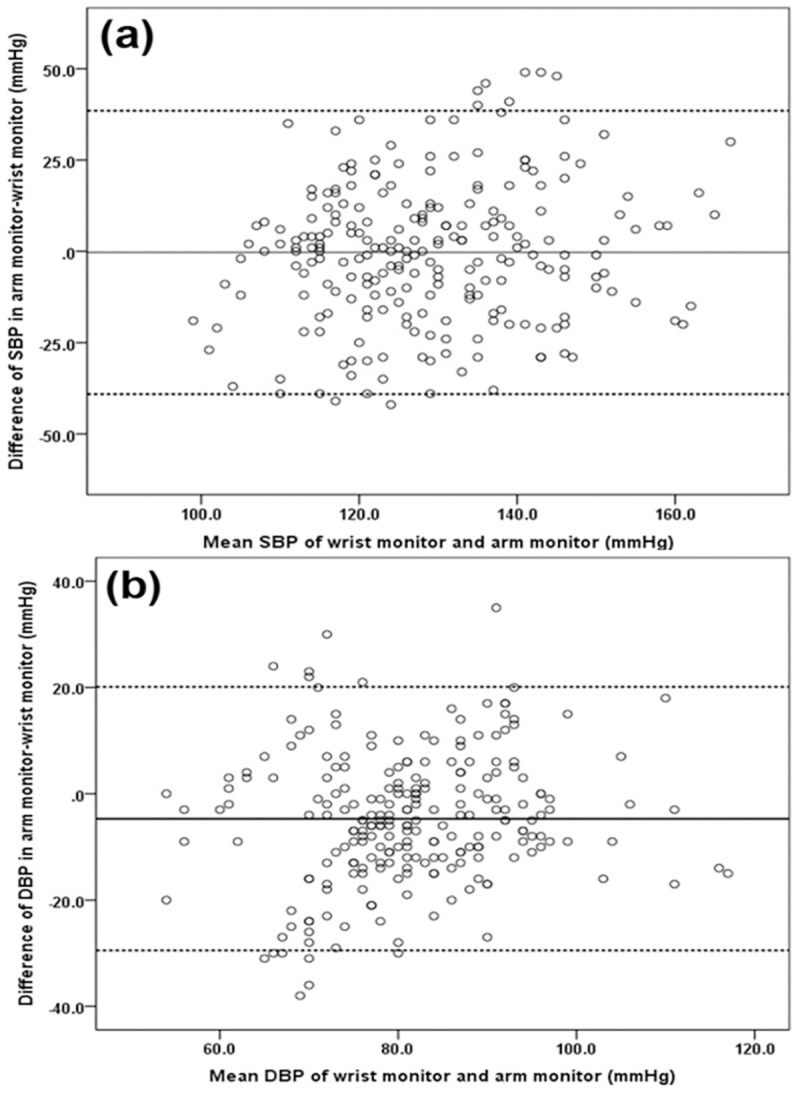
Bland–Altman plots presenting the mean and the difference in blood pressure measurements (mmHg) between the wrist and arm monitors for sleeping SBP (a) and sleeping DBP (b). The solid black horizontal line represents the mean difference and the dotted horizontal lines represent 2SD above and below the mean. There was a mean difference of -0.3 mmHg and a 2SD difference between the arm monitor and the wrist monitor of -39.1to 38.5 mmHg in SBP and a mean difference of -4.7 mmHg and a 2SD difference of -29.5 to 20.1 mmHg in DBP. DBP = diastolic blood pressure, SBP = systolic blood pressure.

The Bland–Altman plot was used to graphically present the difference between each of the wrist and arm measurements plotted against the average of the two measurements. Fig 1 shows the mean of the paired 24-hour SBP and DBP values as Bland–Altman plots. There was a 2SD difference between the arm monitor and the wrist monitor of -37.7 to 39.1 mmHg for SBP and -30.9 to 23.1 mmHg for DBP. There was a systematic difference between the arm monitor and the wrist monitor measurements over the entire range of BP values with the arm monitor showing 0.7 mmHg mean higher SBP values and 3.9 mmHg mean lower DBP values than the wrist monitor. Fig 2 shows the mean of the paired sleeping SBP and DBP values as Bland–Altman plots. There was a 2SD difference between the arm monitor and the wrist monitor of -39.1 to 38.5 mmHg in SBP and -29.5 to 20.1 mmHg in DBP. SBP values by the arm monitor were 0.3 mmHg lower than the wrist monitor. DBP values by the arm monitor were 4.7 mmHg lower than the wrist monitor.

## Discussion

Ambulatory blood pressure monitoring is important in evaluating average 24-hour BP levels, circadian rhythm, sleeping BP and BP variability but many patients are reluctant to use standard upper arm ABPM devices [9, 19–22]. In the present study, we compared two validated ABPM devices for agreement of recordings and acceptability under ambulatory conditions. The success rate of the BP measurements was higher with the arm monitor than the wrist device, probably because the arm monitor is less sensitive to slight movements in the cuff whereas the wrist monitor requires the tonometric sensor to be in stable contact with the radial artery to measure BP and it can easily be displaced in some positions.

A previous study which compared the BPro device with the A&D TM 2431 arm monitor examined BP measurements at different arm positions and found that the BP values provided by the wrist monitor were almost identical in three arm positions and the BP was significantly higher for the wrist monitor than for the arm monitor in the arm-raised position [15].

One of the problems in the use of devices that measure BP at the upper arm is that arm circumference varies and physicians or patients may use cuffs which do not match the arm circumference. The measured BP values will be lower than the true value when a large cuff is used in a slim patient and BP will be higher than the true value when a narrow cuff is used in obese patients so it is important to use the correct cuff size for the patient with arm-type monitors [23–25]. However, this problem is encountered less frequently with wrist-type monitors. Because the wrist size is more similar between obese and non-obese individuals, the wrist-type BP monitor measurements are not affected to such a great extent as arm cuff monitors by body size [26–29].

One disadvantage of the wrist monitor is that when the position sensor moves to an unsuitable position, the BP measurement will fail. In our study, the successful recoding rate with the wrist monitor was considerably lower than with the arm-type device. The success rate of BP measurement with the BPro was as low as 58.9% for 24 hours, 56.2% for the waking period and 66.3% for the sleeping period. However, because the frequency of measurements was greater with the wrist monitor than with the arm monitor during the sleeping period there were more successful readings with the wrist monitor than with the arm monitor at nighttime.

A significant number of patients prefer devices that measure BP at the wrist because of their convenience and they are usually more comfortable to use compared with the arm-cuff type device. The wrist monitor measures BP without compression of the wrist but with slight pressure on the radial artery. In the present study, 10 patients had some discomfort using the wrist monitor. We found this could usually be relieved by adjusting the position or tightness of the wrist monitor and patients could be taught how to do this themselves. Another advantage of the wrist monitor is that there is less noise from the device during the night, which is an important issue in measuring the true BP during the sleeping period.

There was a wide range of differences between readings with the arm monitor and the wrist monitor with a 2SD difference of -37.7 to 39.1 mmHg for 24 hour SBP and -30.9 to 23.1 mmHg for 24 hour DBP (Fig 1). This may be related to errors in the recordings from both devices and also that the measurements were not taken at exactly the same time. Despite these inconsistencies in BP measurements and the systematic overestimation of DBP with the BPro device we would suggest it may be useful in certain patients who find it acceptable as an alternative to an upper arm device to obtain information on the BP variability during 24 hours and the nighttime BP during sleep.

Some studies have shown that mean asleep BP evaluated by ABPM was the most significant predictor of event-free survival in hypertensive patients [8]. High levels of sleeping BP may be best managed by ingestion of at least one hypertension medication at bedtime [9]. In certain subgroups of hypertensive patients, such as those with non-diabetic chronic kidney disease, the nighttime SBP load was found to be an independent predictor of target-organ damage [30]. However, in one study, the ABPM did not improve the ten-year prediction of cardiovascular events compared to office BP and nighttime BP measurements did not improve the prediction compared to daytime measurements [31].

The Hygia Chronotherapy Trial reported that routine ingestion of ≥ 1 antihypertensive medication at bedtime, as opposed to upon waking, was associated with better ABPM control, especially the asleep BP, and with reduced CVD events [32]. However, the findings and advice from this report have been disputed [33] and current guidelines do not recommend routine administration of antihypertensive treatment at bedtime.

Another potential advantage of tonometric devices is that they can be used to estimate central aortic systolic pressure using the N-point moving average method. The BPro device is not only capable of 24-hour ABPM but also for capturing radial arterial waveforms. Results for central aortic systolic pressure from the BPro and SphygmoCor devices in patients with type 1 diabetes showed strong correlations [16].

Central BP changes are thought to have a major influence on the reduction of CVD events with different antihypertensive therapies [34–36]. The evidence and clinical importance of central blood pressure has been discussed in previous reviews [37]. However, a recent analysis of data from 5608 participants enrolled in nine studies, found that CVD end points were not more strongly associated with central BP measurements than with peripheral BP measurements [38].

The present study has several limitations: firstly, the BP values compared for the two devices were not taken at exactly the same time but the time difference was limited to be ≤5 minutes and the heart rate difference between recordings was required to be ≤5 beats/minute to avoid situations when there was a sudden change in activity. Secondly, the number of readings that could be compared between the two devices was limited. Thirdly, although there was a wide range in age of the patients, there were not sufficient very elderly patients to determine if age-related changes in arterial stiffness affected the results.

## Conclusion

The mean SBP values both awake and sleeping showed reasonable agreement for the wrist monitor and the arm monitor but mean DBP were systematically overestimated by about 4 mmHg with the wrist monitor over 24 hours. There were considerable discrepancies for individual readings between the two devices as shown by the Bland–Altman plots. More patients found the wrist monitor more comfortable to use than the arm monitor. The successful recoding rate was considerably lower with the wrist monitor but this could be overcome by having more frequent attempted recordings. Wrist monitors, such as the BPro, may be useful for repeated monitoring to assess changes in 24-hour and sleeping BP which could facilitate adjustment of medications.

## Supporting information

S1 Data (XLS)
